# Molecular Characterization of the *Mycobacterium tuberculosis* Complex in Humans and Cattle

**DOI:** 10.3390/antibiotics15080771

**Published:** 2026-08-10

**Authors:** Jacqueline Samuel Ulomi, Peter M. Mbelele, Jonas Ngowo, David Mtweve, Helena Dela, Bruno Enagnon Lokonon, Bassirou Bonfoh, Esther G. Kimaro, Beatus Lyimo

**Affiliations:** 1 Department of Global Health and Biomedical Sciences, School of Life Sciences, Nelson Mandela African Institution of Science and Technology, Arusha P.O. Box 447, Tanzania; mbelelepeter@yahoo.com (P.M.M.); jonas.ngowo@gmail.com (J.N.); david.mtweve@nm-aist.ac.tz (D.M.); esther.kimaro@nm-aist.ac.tz (E.G.K.); beatus.lyimo@nm-aist.ac.tz (B.L.); 2 Centre Suisse de Recherches Scientifiques en Côte d’Ivoire, Abidjan 01 BP 1303, Côte d’Ivoire; brunolokonon@gmail.com (B.E.L.); bassirou.bonfoh@csrs.ci (B.B.); 3 Kibong’oto Infectious Disease Hospital, Kilimanjaro P.O. Box 12, Tanzania; 4 Department of Microbiology and Immunology, Muhimbili University of Health and Allied Sciences, Dar es Salaam P.O. Box 65001, Tanzania; 5 Noguchi Memorial Institute for Medical Research, Legon, Accra P.O. Box LG 581, Ghana; helena.ogum@gmail.com; 6 Laboratory of Biomathematics and Forest Estimation, University of Abomey-Calavi, Abomey-Calavi 04 BP 1525, Benin

**Keywords:** phylogenetic analysis, lineages, *M. tuberculosis* complex (MTBC), whole-genome sequencing, Manyara

## Abstract

Background/Objectives: Zoonotic tuberculosis (TB) remains a persistent public health challenge worldwide. It is particularly common in settings with close human–livestock–environment interactions. In Tanzania, progress toward TB control is increasingly threatened by multidrug-resistant tuberculosis (MDR-TB), yet genomic data from regions characterized by pastoralist and mining activities remain scarce. This study employed whole-genome sequencing (WGS) to characterize *M. tuberculosis* complex (MTBC) strains circulating among human and cattle populations in the Manyara region of northern Tanzania, with a focus on resistance-associated mutations and phylogenetic relationships. Methods: This cross-sectional study was conducted between September 2024 and February 2025. A total of 178 presumptive human TB cases provided sputum samples. From cattle, 161 samples were collected (110 milk samples and 51 lymph node aspirates), with each animal contributing only one type of sample. Specimen were analyzed using GeneXpert MTB/RIF, Lowenstein–Jensen culture, and WGS. Phylogenetic reconstruction was performed using SNP-based methods and IQ-TREE2 version 2.2.0. Results: Among human participants, 14 (7.8%) sputum samples were GeneXpert positive and were confirmed as members of MTBC by LJ culture. In cattle, one (0.62%) lymph node aspirate was positive for MTBC. Significant predictors of MTBC positivity included previous TB history, weight loss, and occupation involving mining and cattle keeping. WGS of five human isolates identified Lineages 1, 3, and 4. One isolate (Sample 98) harbored mutations associated with XDR-TB. Conclusions: WGS revealed *M. tuberculosis* Lineages 1, 3 and 4 circulating in the Manyara region, with diverse genetic mutations conferring with resistance to first- and second-line anti-TB drugs. These findings highlight the importance of integrated genomic surveillance to monitor drug resistance patterns in Tanzania and similar settings across human and animal populations.

## 1. Introduction

Tuberculosis (TB), caused by members of the *M. tuberculosis* complex (MTBC), is a preventable and generally curable disease; however, it remains the leading cause of mortality from a single pathogen worldwide. For instance, the 2025 World Health Organization (WHO) global report estimated that over 10 million people develop TB annually and that 1.23 million people died in 2024 [1]. The MTBC is a group of species within the Mycobacterium genus with different host preferences and geographical distributions. For example, *M. tuberculosis* predominantly infects humans globally, while *M. africanum* is mainly found in West Africa [2]. Other members include *M. bovis*, which primarily causes TB in cattle; *M. caprae*, which mainly affects goats and other ruminants; *M. microti*, which is primarily found in rodents; and *M. canettii*, which is rare and mainly associated with humans and environmental reservoirs [3]. Together, these species indicate possible transmission at the human–animal interface, known as zoonosis [2].

TB is a highly transmissible airborne infectious disease, primarily spread from individuals with active pulmonary TB to susceptible individuals through inhalation of aerosolized droplets [1]. In addition to human-to-human transmission, zoonotic transmission can occur through close contact with infected livestock, posing a significant risk to pastoralists, farmers, abattoir workers, and veterinarians [4]. The consumption of unpasteurized dairy products, contaminated animal products, and undercooked meat from infected animals further amplifies the risk of transmission [4]. Weak diagnostic and surveillance systems, including inadequate bovine TB surveillance, suboptimal meat inspection practices, and limited integration of One Health approaches within veterinary and public health TB control programs, significantly constrain effective disease detection, monitoring, and control [5]. Furthermore, socioeconomic and environmental factors, including close cohabitation with animals among pastoralist and nomadic communities, overcrowded living conditions, and poor ventilation, further facilitate TB transmission and sustain disease spread within affected populations [6]. These risks are greatest in settings where frequent human–livestock interactions coincide with constrained health systems, further facilitating transmission and contributing to an increased TB burden [5]. Global estimates suggest that zoonotic TB (zTB) accounts for approximately 1–1.5% of the estimated 10 million annual incident human TB cases worldwide [7]. Nonetheless, the true burden remains underestimated due to limited diagnostic capacity and insufficient surveillance systems within national TB programs across both human and animal health sectors, particularly in low- and middle-income countries such as Tanzania, where close contact between humans and livestock is common [8].

In these settings, smear microscopy is widely available for detecting acid-fast bacilli (AFB) across mycobacterial species. However, its sensitivity is limited, particularly in non-sputum specimens such as lymph node aspirates and animal-derived samples like milk, and it cannot differentiate members of the MTBC [9]. Culture is the gold standard for isolating mycobacterial species, yet it is time-consuming, often delaying results for up to eight weeks. It also requires sophisticated infrastructure, including biosafety level 3 (BSL-3) containment facilities, to protect laboratory personnel and the surrounding community [10]. In addition, optimized procedures are needed for the recovery of *M. bovis* from clinical and animal specimens [11,12]. In the past two decades, since the early 2000s, WHO has endorsed molecular diagnostic tests, including GeneXpert^®^ MTB/RIF and line probe assays (GenoType MTBDRplus/sl), for the detection of members of the MTBC and resistance to anti-TB drugs such as rifampicin, isoniazid, fluoroquinolones, and injectable aminoglycosides (now largely obsolete in TB care) [13]. However, these methods have limited ability to distinguish closely related MTBC species and to assess susceptibility to key anti-TB treatment options, including pyrazinamide, ethambutol, bedaquiline, delamanid, cycloserine, and clofazimine. These limitations reduce the accuracy of zoonotic TB burden estimates, mainly caused by *M. bovis*, and obscure transmission dynamics between humans and animals, among other constraints of TB care.

Whole-genome sequencing (WGS) overcomes many of these limitations by providing high-resolution identification of MTBC species, strain differentiation, detection of subtle genetic variations, resolution for lineage classification, resistance profiling, and phylogenetic inference [14]. This makes it a powerful tool to map interspecies transmission and inform targeted control strategies. However, its use remains largely confined to research settings in many low- and middle-income countries due to the high costs associated, which further limits understanding of the true burden of zoonotic TB, genomic diversity, transmission patterns, and potential interspecies spread of strains particularly in resource-constrained settings with close human–animal interaction such as Tanzania as described by [15]. This study used WGS to characterize MTBC strains isolated from humans and integrated with socio-demographic, clinical, lifestyle, and transmission risk profiles. This approach improved understanding of MTBC species distribution, genetic diversity, and drug resistance patterns, as well as potential zoonotic transmission pathways. These insights are essential for informing integrated and strengthening diagnostics, surveillance, and targeted interventions in high-burden settings.

## 2. Results

### 2.1. Socio-Demographic and Clinical Characteristics of Human Participants

Of the 339 samples, 178 (52%) were collected from humans and 161 (48%) from cattle. In human participants, 161 (90.4%) of the 178 were male, and 103 (57.8%) were engaged in mining and cattle keeping. The mean age was 37.6 (SD 10.9) years. Additionally 120 (67.4%) reported a persistent cough lasting more than two weeks, 170 (95.5%) reported living with persons with a persistent cough, 158 (88.7%) had prior contact with a person with active TB, and 36 (20.2%) reported a previous history of TB treatment (Table 1).

### 2.2. Clinical Characteristics of Cattle

Among the 161 cattle, 135 (83.8%) exhibited general weakness and emaciation, 67 (41.6%) had enlarged lymph nodes, 65 (40.4%) had a cough lasting more than two weeks, and 115 (71.4%) had experienced weight loss (Table 2).

### 2.3. M. tuberculosis Complex Detection in Xpert^®^ MTB/RIF Assay and LJ Culture

Of the 339 participants (178 humans and 161 animals), 15 (4.4%) tested positive for MTBC by GeneXpert MTB/RIF and/or Lowenstein–Jensen (LJ) culture, including 14/178 (7.9%) humans and 1/161 (0.6%) of cattle (Table 3).

### 2.4. Detection of M. tuberculosis Lineages and Their Phylogenetic Relationships in Cultured Isolates

**Figure 1:**
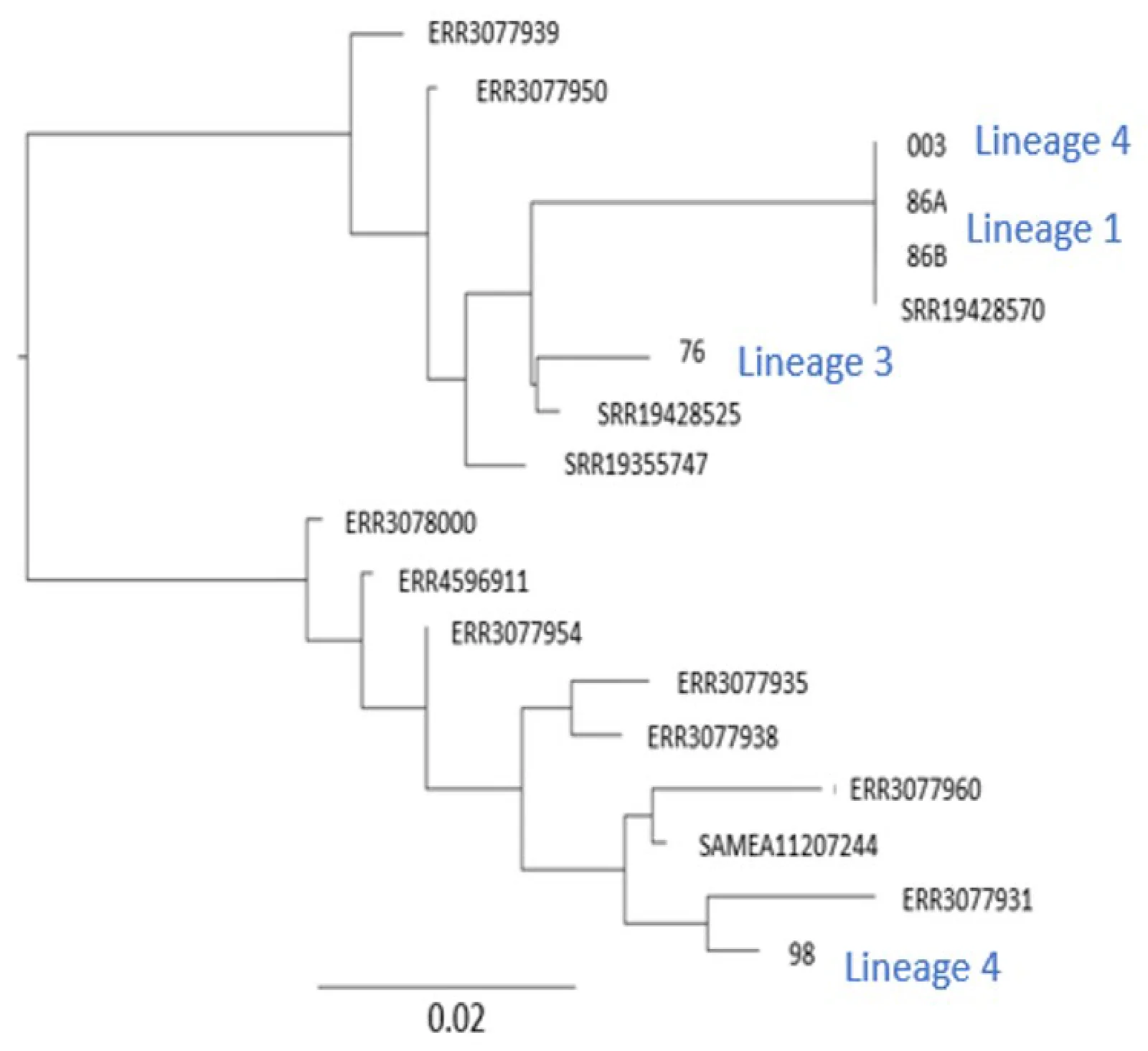
Maximum likelihood phylogenetic tree reconstructed from whole-genome SNP data of *M. tuberculosis* isolates using IQ-TREE2 v2.2.0.

All five cultured isolates that underwent WGS were confirmed as *M. tuberculosis*. Neither *M. bovis* nor other animal-associated MTBC species were detected. The phylogenetic tree demonstrated clear clustering of *M. tuberculosis* isolates into Lineage 1 (Indo–Oceanic, n = 2), Lineage 3 (East African–Indian, n = 1) and Lineage 4 (Euro-American, n = 2), with consistent topology observed across analyses. Details of the lineages are provided in Table 4 and Figure 1.

### 2.5. Whole Genome Drug Resistance Prediction

The *M. tuberculosis* isolates exhibited diverse mutations conferring resistance to first-line anti-TB drugs used for drug-susceptible TB, as well as second-line drugs used for drug-resistant TB in Tanzania and elsewhere. For example, isolate 003 (belonging to the lineage Euro-American) harbored a p. Trp119 mutation in the *pncA* gene associated with pyrazinamide resistance. Isolate 76 (belonging to the East African–Indian lineage) carried a Gln10 mutation in the *pncA* gene. Isolates 86A and 86B were two different isolates obtained from different individuals, and WGS identified both isolates as belonging to the Indo–Oceanic lineage; n = 2 both harbored a c.-777C>T mutation in the *inhA* gene associated with resistance to isoniazid and ethionamide. Interestingly, isolate 98 carried mutations in multiple genes conferring resistance to both first- and second-line anti-TB drugs, consistent with extended drug-resistant tuberculosis (XDR-TB). Details of specific mutations and types of genetic variation in genes associated with resistance to isoniazid, rifampicin, ethambutol, pyrazinamide, fluoroquinolones (moxifloxacin and levofloxacin), bedaquiline, cycloserine, ethionamide, clofazimine, and streptomycin are presented in Table 4. The identified variants included missense mutations, stop-gained mutations, frameshift mutations, and upstream regulatory variants.

### 2.6. Risk Factors for TB Infection in the Human

In the univariable Firth logistic regression analysis (Table 5), occupation, weight loss, history of tuberculosis, fever for at least two weeks, cough for at least two weeks, and night sweats were significantly associated with MTBC positivity (*p* < 0.05). Contact with a tuberculosis patient met the criterion (*p* < 0.25) for consideration in the multivariable analysis, whereas age, district, contact with cattle with tuberculosis, coughing blood, and living with a person with persistent cough were not significantly associated with MTBC positivity.

In the multivariable analysis, following purposeful variable selection, fever for at least two weeks, night sweats, contact with a tuberculosis patient, and cough for at least two weeks were sequentially removed because they were not independently associated with MTBC positivity and their removal did not materially change the regression coefficients of the remaining variables (change in estimate <15%). Occupation, weight loss, and history of tuberculosis remained independently associated with MTBC positivity (Table 6). Compared with cattle keepers, participants engaged in both mining and cattle keeping had approximately five-fold higher odds of MTBC positivity (aOR = 5.28, 95% CI: 1.30–31.45; *p* = 0.018). Participants reporting weight loss had 4.83 times higher odds of MTBC positivity than those without weight loss (aOR = 4.83, 95% CI: 1.45–18.34; *p* = 0.010). Likewise, individuals with a previous history of tuberculosis had four-fold higher odds of MTBC positivity compared with those without a history of tuberculosis (aOR = 4.01, 95% CI: 1.21–13.81; *p* = 0.023).

## 3. Discussion

This cross-sectional study applied WGS to characterize the population structure of MTBC and their associated drug resistance patterns in human and cattle in the Manyara region. The analysis provides an understanding of interrelated transmission dynamics in pastoralist settings, including whether it is predominantly zoonotic or human-driven. It also evaluates whether genomic evidence of resistance aligns with or extends beyond what is detected by routine diagnostics. The use of WGS in developed countries as a diagnostic and disease surveillance tool is slowly gaining traction in low-resource and high-tuberculosis burden settings [16]. Employing WGS in this study not only fills a serious gap in the existing literature about the Manyara region but also showcases the possibility of progressive genomic techniques in enhancing our understanding of TB dynamics in resource-constrained settings.

Risk factor analysis identified that previous history of TB, weight loss and occupation involving mining and cattle keeping as independent predictors of MTBC positivity after adjustment for potential confounders. These findings are consistent with report from India showing that weight loss in TB patients due to undernutrition being a driver of TB, which highlight relapse, reinfection, or incomplete treatment as drivers of community burden [17]. These findings suggest two distinct pathways: mining-related occupational exposure that enhances host vulnerability, and cattle-associated interfaces that facilitate MTBC transmission within agro-pastoral environments [18,19]. Although the LJ culture of one cattle sample was positive for MTBC, the DNA concentration was insufficient for sequencing, but the observed pattern still points to cross-host MTBC circulation within a One Health context in the study area, as previously described in southern Ethiopia, where zoonotic TB has been reported to be sporadic [5]. The lack of a significant association between infection and reported contact with cattle TB or living with coughing individuals suggests that transmission is likely shaped more by indirect, community-wide and occupational exposures than by direct household or animal contact alone. This aligns with earlier findings from northern Tanzania, where similar exposure variables have shown weak or inconsistent associations with TB outcomes, largely attributed to reliance on self-reported contact histories and the dominant role of broader, unrecognized transmission networks in high-burden settings [4].

We found that the genotypic drug resistance among the five positive sequenced isolates indicates resistance extending beyond the standard first-line regimens, which include isoniazid, rifampicin, ethambutol, and pyrazinamide, to include some second-line drugs such as fluoroquinolones (levofloxacin and moxifloxacin), bedaquiline, clofazimine, and ethionamide. This signifies the circulation of highly resistant strains with limited treatment options. Mutations and variation types (Table 4) in the genes associated with these drugs are well-established markers of high-level resistance and strongly predict phenotypic resistance [20]. Our analysis further revealed missense, stop-gained, frameshift, and upstream_gene_variant types of variation, each contributing differently to how *M. tuberculosis* evades treatment. The predominance of missense mutations, such as those in *katG*, *rpoB*, *embB*, *mmpR5* and *gyrA*, aligns with findings from Tanzania [21], where these mutations are well documented as key drivers of high-level resistance to isoniazid, rifampicin, ethambutol and moxifloxacin, reinforcing their central role in MDR-TB evolution by either impairing activation of the drug or affecting the beta subunit of RNA polymerase. Moreover, the detection of upstream gene variants, particularly in the *inhA* promoter region, indicates an additional resistance mechanism through altered gene expression rather than structural protein changes, a mechanism that reduces isoniazid drug binding and leading to low-level resistance to isoniazid drug. This is also reported in East African settings and associated with cross-resistance between isoniazid and ethionamide [22]. The identification of frameshift mutations, such as duplications in *mmpR5*, suggests disruption of regulatory pathways controlling efflux pumps, which have increasingly been linked to reduced susceptibility to drugs like clofazimine and bedaquiline. Additionally, the presence of stop-gained mutations points to potentially severe functional loss of key genes, a mechanism widely associated with pyrazinamide resistance through disruption of *pncA*. Similar resistance patterns have been reported in Tanzania [23], Zambia [24], Egypt [25], and other neighboring countries, where molecular studies documented MDR and pre-XDR tuberculosis strains carrying mutations in the same gene regions. These findings underscore the silent and growing challenge of drug-resistant TB in the region and highlight the importance of routine genomic surveillance to guide appropriate therapy and limit the spread of difficult-to-treat strains [14]. Some mutations in *rpoB*, *katG*, and the *inhA* promoter region, including both missense and upstream variants, were not detected by the Xpert MTB/RIF assay. Due to its design, which targets only the 81 bp rifampicin resistance-determining region (RRDR) of the *rpoB* gene using specific probes, mutations outside this region or those that do not significantly impact probe binding can be missed [26]. WGS, however, captures a broader spectrum of genetic variation, including rare and low-frequency mutations across multiple resistance-associated genes. Similar observations have been reported in studies from Tanzania [23], South Africa [27], and Zambia [24] where sequencing revealed resistance-conferring mutations undetected by Xpert diagnostics, demonstrating the added value of genomic approaches in comprehensively characterizing drug resistance patterns in high-burden settings. These results increase the potential of WGS as a molecular tool to complement other tests like Gene Xpert^®^ [23].

WGS revealed the predominance of Lineage 4 in human isolates which aligns with regional patterns in east and southern Africa, where this Euro-American lineage is frequently associated with multidrug-resistant TB (MDR-TB) and XDR-TB [27,28]. The detection of an XDR isolate for sample 98 in a rural area like Manyara is particularly concerning, as it may indicate undetected transmission chains facilitated by human mobility, trade, and occupational factors such as mining-related silica dust exposure [19]. Lineage 3 (East African–Indian) isolates, including one with pyrazinamide resistance, reflect endemic transmission rather than recent outbreaks, consistent with lower drug resistance prevalence in this lineage [29]. Although based on these five samples, the findings underscore clear differences in the resistance mutation profiles between the circulating Lineage 1, 3 and 4 in the study area.

Phylogenetic analysis, including a 5-SNP unmasked tree, demonstrated close genetic relatedness among certain Lineage 4 samples with short branch lengths, suggesting recent common ancestry or shared exposure. Sample 76 clustering with Lineage 3.1.1 and publicly available genome SRR19428525 further supports evolutionary ties to East African–Indian strains in the region [21]. Sample 98 clustered closely with the publicly available genome ERR3077931 [23], while samples 003, 86A, and 86B grouped with SRR19428570 [21], suggesting that these isolates share a close evolutionary relationship and may have originated from related transmission networks and infection or common ancestral strains. This clustering pattern is consistent with previous studies showing that MTBC strains within the same lineage tend to group together due to shared genetic characteristics and regional circulation patterns. The presence of Lineage 1 and Lineage 4 strains in this study further reflects the known diversity of MTBC circulating in East Africa, particularly in northern Tanzania, where multiple lineages have previously been reported [23].These genomic patterns are robust to SNP masking, and underscore the value of WGS in distinguishing true biological signals from sequencing artifacts, though SNP-based transmission inferences require integration with epidemiological data.

Limitations of this study include the relatively small number of samples analyzed by whole-genome sequencing and the cross-sectional design, which constrain the generalizability of the findings and preclude determination of the direction of transmission between humans and cattle. In addition, drug resistance was predicted from genomic mutations without phenotypic drug susceptibility testing, and environmental and wildlife sources were not included. Despite these limitations, the study provides valuable baseline information on circulating *M. tuberculosis* complex strains and resistance-associated mutations at the human–cattle interface, which can guide future One Health studies with larger sample sizes and longitudinal follow-up.

## 4. Materials and Methods

### 4.1. Study Design

This cross-sectional study was conducted between September 2024 through February 2025 to understand the characteristics of the *M. tuberculosis* complex and identify their potential factors for transmission in human and cattle population with drug-susceptible tuberculosis.

### 4.2. Study Settings

**Figure 2:**
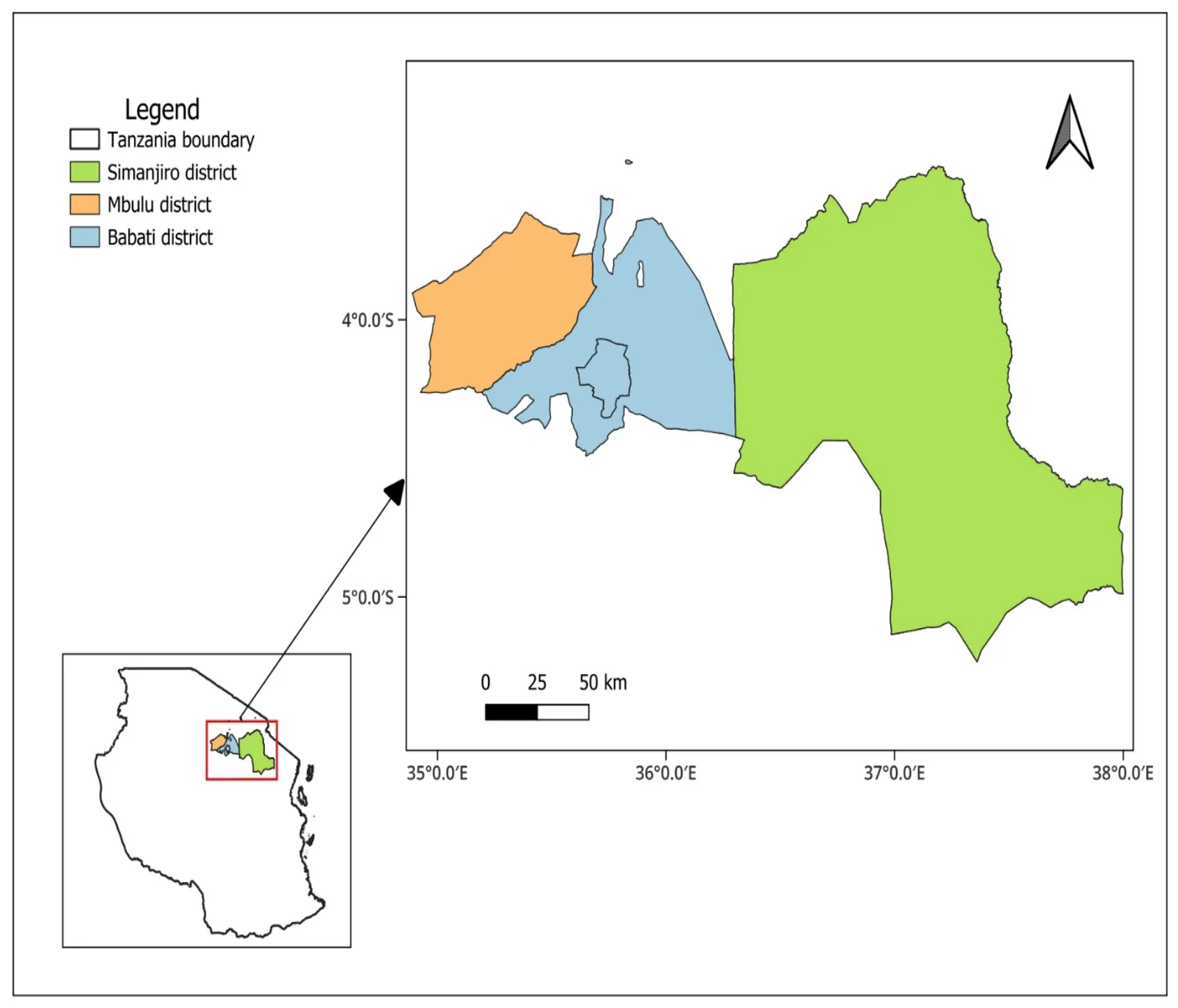
Map of the study area showing districts of the Manyara region, Tanzania where the samples were taken from.

The study population was recruited in the Manyara region of northern Tanzania, specifically in Simanjiro District (including Mirerani), Babati Town (including Dongobesh and Sigino), and Mbulu District (Figure 2). The region is characterized by pastoralism, mining activities in the Mirerani area, and agricultural practices, particularly livestock keeping, all of which promote close human–livestock interactions and TB transmission [19]. Sputum samples were tested for tuberculosis using the Xpert^®^ MTB/RIF assay at Mirerani Health Center, Simanjiro District Hospital, and Mrara Hospital in Babati Town. Isolation of the *M. tuberculosis* complex was performed, using LJ enriched with both glycerol and pyruvate culture methods, at the Kibong’oto Public Health Laboratory, located within Kibong’oto Infectious Diseases Hospital in the Kilimanjaro region, Tanzania. The Kibong’oto Public Health Laboratory is a biosafety level 3 facility and serves as a zonal reference laboratory for tuberculosis, supporting regions in northern Tanzania, including Manyara.

### 4.3. Study Participants

This study enrolled adult male and female (≥15 years) presenting with presumptive pulmonary TB in the Manyara region. Presumptive TB was defined as the presence of clinical signs and symptoms suggestive of TB, including a cough lasting at least 2 weeks (with or without blood-streaked sputum), chest pain, shortness of breath, weight loss of at least 10 kg within 3 months, fever, excessive night sweats, and/or loss of appetite. Participants were included if they were able to provide sputum samples and gave informed consent. Pregnant women and participants who were on anti-TB treatment and within the preceding 7 days were excluded due to potential compromise of sputum sample quality and to avoid interference with the performance of the diagnostic tests. The study also included cattle from participating households and around the environment that presented with signs suggestive of tuberculosis, such as chronic cough, emaciation, lethargy, anorexia, low-grade fever, or enlarged lymph nodes, as assessed by a district veterinarian. Cattle were included if the household consented to the collection of lymph node aspirates and milk samples for TB testing. Cattle receiving antibiotic treatment without clinical signs were excluded. Animals or humans, from whom suitable samples could not be obtained, were also excluded.

### 4.4. Sample Size

The minimum sample size for human and cattle participants was calculated using Cochran’s formula [30] with an estimated pulmonary tuberculosis prevalence of 8% for the human sample, as previously reported by Mbuya et al. [19], and of 13% for the cattle sample as reported by Katale et al. [31]. Assuming a 95% confidence interval and a 5% margin of error, the minimum required sample size for human participants was 114. However, to improve statistical precision and account for potential losses due to non-response, a total of 178 human and 161 cattle participants were enrolled in the study.

### 4.5. Sample Collection, Transportation and Handling

A total of 178 human participant provided at least 5 mL into a 50 mL falcon tube of duplicate sputum sample for detecting MTBC complex in Xpert^®^ MTB/RIF ultra, and for isolating it in LJ culture enriched with both glycerol and pyruvate as per manufacturer instruction. For cattle participants, 5 mL of 110 raw milk samples and at least 3 mL of 51 lymph node aspirates were obtained in a 15 mL falcon tube with veterinary assistance. Milk sample was collected from lactating cattle in a herd with history of TB infection but no signs of palpable enlargement of the superficial lymph node. Lymph node aspirate sample was collected from cattle with palpable enlargement of superficial lymph node, chronic weight loss leading to emaciation, persistent cough, suspected generalized or localized TB lesions, and showing systemic signs without obvious milk abnormalities. All samples were transported under cold chain conditions (2–8 °C) to the Microbiology Laboratory at Kibong’oto Infectious Diseases Hospital in the Kilimanjaro region. Upon arrival, human samples were stored at 2–8 °C, while cattle samples were stored at −20 °C pending further laboratory analysis [32].

### 4.6. Laboratory Procedures

#### 4.6.1. Gene Xpert Analysis

The sputum samples were tested for TB using Xpert^®^ MTB/RIF assay in accordance with the manufacturer’s instruction and described in previous studies [33,34]. In brief, the sputum was homogenized and liquefied by treating with the sample reagent at a 2:1 ratio (reagent: sputum), followed by vigorously shaking for 10 s, and incubated at room temperature for 15 min. A 2 mL of homogenized sputum was transferred into the Xpert^®^ cartridge and the Xpert^®^ MTB/RIF module to continue with automatic DNA extraction, amplification and detection of MTBC. The MTBC detection was done by amplifying specific DNA sequences of the *rpoB* gene probed with five molecular beacons A, B, C, D and E, each labeled with a unique fluorophore. During analysis, the maximum valid quantification cycle (Cq) was 39.0 for Probes A–C and 36.0 for Probes D–E. The test was positive for MTBC when at least two probes showed Cq values within the valid range with a delta Cq < 2.0. Bacterial load was semi-quantitatively classified as very low (28.0–40), low (20.5–27.9), medium (18.5–20.4), or high (15–18.4). A trace result was accepted when detection was based only on IS1081 and IS6110 targets. The assay was considered negative for MTBC when one or no probe was positive [35].

#### 4.6.2. Lowenstein Jensen Culture and Identification

Sputum from human and lymph node aspirate from cattle were decontaminated using the modified Petroff 4%NaOH method [36]. Milk sample was decontaminated with addition of a drop of 0.05% phenol red indicator into a sediment of milk suspended with 2 mLs of sterilized normal saline after centrifugation for 15 min at 3000 rpm, followed by the addition of an equal volume of sterilized 4-N sodium hydroxide and sterile 4-N hydrochloric acid [37]. Culture on LJ medium was performed following Clinical and Laboratory Standards Institute (CLSI) guidelines and SOPs at Kibong’oto Public Health Laboratory. Briefly, 200 µL of sputum sediment was inoculated onto LJ slopes supplemented with glycerol for detection of *M. tuberculosis* and pyruvate for M. bovis. Quality control was ensured using the reference strain *M. tuberculosis* H37Rv (ATCC 27294) as a positive control and distilled water as a negative control [38]. Inoculated LJ media were incubated aerobically at 37 °C and observed weekly for up to 8 weeks before being declared negative. Culture-positive isolates were confirmed as acid-fast bacilli (AFB) using LED fluorescent microscopy and as members of the MTBC using rapid MPB64 antigen detection assay following manufacturer instructions [39].

#### 4.6.3. Genomic DNA Extraction

Genomic DNA was extracted from the pure 15 cultured isolates using Qiagen DNeasy Blood and Tissue Kit (Hilden, Germany) following the manufacturer’s instructions. The method has been previously used to extract DNA of *M. tuberculosis* complex for molecular characterization of TB as described by [40]. Briefly, 15 cultured MTBC isolates from individual samples were heat-killed at 96 °C. The cultured cells were lysed with proteinase K and Buffer AL, followed by incubation at 56 °C. Ethanol was then added to the mixture and loaded onto a spin column, where DNA binds to the membrane during centrifugation. The column was washed sequentially with Buffer AW1 and AW2 to remove impurities. Finally, DNA was eluted using Buffer AE. The DNA concentration and quality were assessed using a Nanodrop spectrophotometer (Thermo Fisher Scientific, Waltham, MA, USA) as per the manufacturer’s instructions. Samples with A260/A280 ratios between 1.8 and 2.0 and A260/A230 ratios above 2.0 in the Nanodrop spectrophotometer were considered acceptable for other molecular analysis. The obtained genomic DNA were 25 μL, shipped in screw-capped cryo-vials and sealed with parafilm to BGI Tech Solutions Hong Kong Co., Limited (Hong Kong, China), for whole-genome sequencing and bioinformatic analysis.

#### 4.6.4. Whole-Genome Sequencing

Whole-genome sequencing was performed on 5 randomly selected DNA samples that met the required DNA concentration and quality thresholds for sequencing. The MGI-compatible DNA library preparation kit from MGI Tech, Shenzhen, China was used to prepare libraries from gDNA following the manufacturer’s protocol as described by [41]. In brief, the genomic DNA of 5 cultured isolates were fragmented, and purified using magnetic beads and enzymatic end-repair to produce blunt-ended DNA and was ligated using platform-specific adapters required for sequencing. For PCR-based libraries, limited amplification enriched adapter-ligated fragments, whereas PCR-free libraries proceeded directly to quality control. The resulting libraries were evaluated for DNA concentration and fragment size distribution to ensure they met sequencing specifications before loading onto the DNBSEQ platform. The WGS was performed at BGI Tech Solutions Hong Kong Co., Ltd. using the DNBSEQ platform (MGI Tech, Shenzhen, China), which generated paired-end reads of 150 bp.

#### 4.6.5. Bioinformatics Analysis

The FASTQ files (raw sequencing data) were quality-filtered using SOAPnuke software version 2.2.1 remove adapter sequences, contaminants, low-quality reads, and short fragments (<150 bp), as previously reported [42]. Reads containing ≥1% ambiguous bases, ≥40% low-quality bases (Q < 20), or ≥50% adapter sequence was discarded. Clean reads encoded in Phred + 33 format were subjected to quality assessment using FASTQC version 0.11.9. Reads were mapped to the *M. tuberculosis* H37Rv laboratory reference strain (ATCC 27294) [43] using Burrows–Wheeler alignment version 0.7.17. Taxonomic confirmation, variant calling, lineage assignment, mapping statistics and variant annotations generated using TB-Profiler version 6.6.3. Alignment quality was further evaluated with Qualimap version 2.2.2, and drug-resistance-associated mutations were predicted using TB-Profiler version 4.4.2. Only sequences meeting quality thresholds of ≥95% genome mapping and ≥30× mean depth were included in the final analysis.

#### 4.6.6. Phylogenetic Tree Construction

Phylogenetic reconstruction was performed using SNP-based methods from 18 genomes (Figure 1), including five study isolates and 13 publicly available *M. tuberculosis* genomes used for contextual comparison. Five samples from this study (003, 86A, 86B, 76, and 98) were analyzed alongside 13 publicly available genomes listed in Table 7 to construct a whole-genome SNP-based phylogenetic tree and assess the genetic relationships among the *M. tuberculosis* isolates [44]. Variant calling was performed using BCFtools version 1.23 to identify single nucleotide polymorphisms (SNPs) and insertion–deletion events (indels). During variant filtering, SNPs located within repetitive genomic regions were removed to minimize false-positive calls resulting from ambiguous read alignment. A concatenated SNP alignment was subsequently generated and used to infer a maximum likelihood phylogenetic tree with IQ-TREE2 version 2.2.0, applying 1000 bootstrap replicates to evaluate branch support. The resulting tree was then visualized and refined using Interactive Tree of Life (iTOL) version 7.42.

### 4.7. Statistical Analysis

Associations between potential risk factors and MTBC positivity were first evaluated using univariable Firth penalized logistic regression to obtain crude odds ratios (cORs), 95% confidence intervals (CIs), and *p*-values. Variables with *p* < 0.25 in the univariable analysis, together with clinically relevant variables, were considered for multivariable analysis following the purposeful selection strategy described by [46]. Because only 15 participants were positive for MTBC and evidence of sparse data was observed, multivariable analysis was performed using Firth penalized logistic regression to reduce small-sample bias and address quasi-complete separation. Non-significant variables were removed sequentially if their exclusion did not change the regression coefficients of the remaining variables by ≥15%, indicating the absence of confounding. Adjusted odds ratios (aORs) with 95% CIs were reported, and statistical significance was assessed at *p* < 0.05.

## 5. Conclusions

This study identified MTBC as the causative agent of tuberculosis in humans and cattle using Xpert^®^ MTB/RIF and LJ culture. Mining and cattle-keeping occupations, weight loss, and previous TB history were independently associated with MTBC positivity. These findings suggest that occupational exposure, clinical vulnerability, and previous infection may contribute to MTBC transmission and persistence. Whole-genome sequencing of five MTBC isolates confirmed *M. tuberculosis* belonging to Lineage 1 (Indo–Oceanic), Lineage 3 (East African–Indian), and Lineage 4 (Euro-American). The isolates harbored diverse genetic mutations associated with resistance to first-line drugs used for drug-susceptible TB treatment and second-line drugs used for drug-resistant TB management. These findings highlight the importance of surveillance for monitoring drug resistance patterns in Tanzania and similar settings across human and animal populations.

## Figures and Tables

**Table 1:** Demographic and clinical characteristics of human participants (*N* = 178).

Variable(s)	Description	Frequency	Percentage (%)
Age group (Yrs)	15–25	24	13.5
	26–36	60	33.7
	37–47	58	32.5
	48–60	36	20.2
Gender	Male	161	90.4
	Female	17	9.5
Mining Worker and cattle keeping	Yes	103	57.8
	No	75	42.2
Cattle keeping	Yes	71	39.9
	No	107	60.1
Family member to cattle owner	Yes	1	0.5
	No	177	99.5
Family member to mining worker	Yes	2	1.1
	No	176	98.8
Former mining worker	Yes	1	0.5
	No	177	99.5
Cough for more than 2 weeks	Yes	120	67.4
	No	58	32.5
Cough blood	Yes	5	2.8
	No	173	97.2
Fever for more than 2 weeks	Yes	44	24.7
	No	134	75.3
Night sweat	Yes	34	19.1
	No	144	80.8
Weight loss	Yes	64	35.9
	No	114	64.0
Contact with TB infected patient	Yes	158	88.7
	No	20	11.2
Living with person with persistent coughing	Yes	170	95.5
	No	8	4.4
History of TB	Yes	36	20.2
	No	142	79.7
Contact with infected TB cattle	Yes	1	0.5
	No	177	99.4

**Table 2:** Clinical characteristics of cattle participants (*N* = 161).

Variable	Description	Frequency	Percentage (%)
Cattle’s weight loss (Kg)	Yes	115	71.4
	No	46	28.6
Cattle’s lymph node enlargement	Yes	67	41.6
	No	94	58.4
Cattle coughing	Yes	65	40.4
	No	96	59.6
Cattle’s general weakness	Yes	135	83.8
	No	26	16.1

**Table 3:** Diagnostic work flow and Identification of MTBC.

Group	Sample	Xpert (+)	Xpert (−)	LJ Culture (+)	LJ Culture (−)
Human	178	14 (7.9%)	164 (92.1%)	14 (7.9%)	164 (92.1%)
Cattle	161	-	-	1 (0.62%)	160 (99.4)
Total	339	14	164	15	324

**Table 4:** Showing *M. tuberculosis* Lineage with association of specific drug resistance (*N* = 5).

Sample Number	*M. tb* Lineage	*M. tb* Sub Lineage	Lineage Name	Drug Resistance	Gene	Mutation of the Gene Affected	Type of Variation
003	4	4.8	Euro-American	Pyrazinamide	*pncA*	p.Trp119	Stop-gained
76	3	3.1.1, 3.1	East African–Indian	Pyrazinamide	*pncA*	Gln10	Stop-gained
86A	1	1.1, 1.1.2	Indo–Oceanic	Isoniazid	*inhA*	c.-777C>T	upstream_gene_variant
				Ethionamide	*inhA*	c.-777C>T	
86B	1	1.1, 1.1.2	Indo–Oceanic	Isoniazid	*inhA*	c.-777C>T	upstream_gene_variant
				Ethionamide	*inhA*	c.-777C>T	
98	4	4.2.2, 4.2.2.2, 4.2	Euro-America	Isoniazid	*katG*	Ser315Thr	Missense
				Rifampicin	*rpoB*	Gln432Glu	Missense
				Ethambutol	*embB*	Met306Leu	Missense
				Pyrazinamide	*pncA*	Glu111	Stop-gained
				Streptomycin	*rpsL*	Lys88Met	Missense
				Clofazimine	*mmpR5*	198dupG	Frame shift
				Cycloserine	*ald*	436_437dupGC	Frame shift
				Moxifloxacin	*gyrA*	Asp94Gly	Missense
				Levofloxacin	*gyrA*	Asp94Gly	Missense
				Bedaquiline	*mmpR5*	198dupG	Frame shift

**Table 5:** Univariate Logistic regression for assessing factors associated with TB infection (N = 178).

Predictor Variable	Variable Category	cOR	95% CI	*p*-Value
Occupation				0.025
	Cattle keeping	1	Reference	
	Mining Worker and Cattle keeping	4.03	1.17–20.99	0.025 **
Weight loss				0.002
	No	1	Reference	
	Yes	5.33	1.81–18.53	0.002 **
History of TB				<0.001
	No	1	Reference	
	Yes	7.00	2.42–21.52	<0.001 **
Fever ≥ 2 weeks				0.002
	No	1	Reference	
	Yes	5.53	1.86–16.21	0.002 **
Age (years)	Continuous	1.01	0.96–1.06	0.769
Contact with cattle with TB				0.497
	No	1	Reference	
	Yes	3.52	0.02–68.88	0.497
Contact with TB patient				0.201
	No	1	Reference	
	Yes	4.40	0.55–569.17	0.201
Cough ≥ 2 weeks				0.002
	No	1	Reference	
	Yes	17.03	2.22–2188.04	0.002 **
Coughing blood				0.964
	No	1	Reference	
	Yes	0.94	0.01–8.91	0.964
District				0.406
	Babati Kijijin	1	Reference	
	Babati Mjini	0.48	0.00–93.63	0.726
	Babati Vijijini	5.00	0.02–1147.15	0.474
	Simanjiro	1.87	0.21–246.72	0.647
Living with person with persistent coughing				0.705
	No	1	Reference	
	Yes	1.68	0.19–221.53	0.705
Night sweat				0.008
	No	1	Reference	
	Yes	4.41	1.49–12.91	0.008 **

** Significant value, cOR is the crude odds ratio computed in this univariate logistic regression.

**Table 6:** Multivariate logistic regression for infection (N = 178).

Variable	Variable Category	aOR	95% CI	*p*-Value
Occupation				0.018
	Cattle keeping	1	Reference	
	Mining Worker and Cattle keeping	5.28	1.30–31.45	0.018 **
Weight loss				0.010
	No	1	Reference	
	Yes	4.83	1.45–18.34	0.010 **
History of TB				0.023
	No	1	Reference	
	Yes	4.01	1.21–13.81	0.023 **

** Significant value, aOR is the adjusted odds ratio computed in this multivariate regression model; CI is confidence interval.

**Table 7:** Accession number and their reference.

S/N	Accession Number	Source Sample	Reference
1	ERR3077939	Human	[23]
2	ERR3077950	Human	[23]
3	SRR19428570	Human	[21]
4	SRR19428525	Human	[21]
5	SRR19355747	Human	[21]
6	ERR3078000	Human	[23]
7	ERR4596911	Human	[23]
8	ERR3077954	Human	[23]
9	ERR3077935	Human	[23]
10	ERR3077938	Human	[23]
11	ERR3077960	Human	[23]
12	SAMEA11207244	Human	[45]
13	ERR3077931	Human	[23]

## Data Availability

The demographic data generated and analyzed during this study are not publicly available due to ethical and confidentiality restrictions, but they can be available from the corresponding author upon reasonable request and permission from Nelson Mandela African Institution of Science and Technology. The raw sequenced data (FASTQ files) will be available in NCBI under project accession number PRJNA1449723 with the reviewer link (https://dataview.ncbi.nlm.nih.gov/object/PRJNA1449723?reviewer=3h6olmadjj73o4s7c6ea0gnudt, accessed on 28 May 2026).

## Abbreviations

The following abbreviations are used in this manuscript

TB	Tuberculosis
MDR	Multi drug resistance
MTBC	Mycobacterium tuberculosis complex
WGS	Whole genome sequence
LJ	Lowenstein–Jensen
XDR	Extended drug resistance
AFB	Acid-fast bacilli
CLSI	Clinical and laboratory standard Institution
SNP	Single nucleotide polymorphism
